# Ethylene, a Hormone at the Center-Stage of Nodulation

**DOI:** 10.3389/fpls.2015.01121

**Published:** 2015-12-22

**Authors:** Frédérique C. Guinel

**Affiliations:** Department of Biology, Wilfrid Laurier University, Waterloo, ONCanada

**Keywords:** model legumes, rhizobia, *sickle*, host immunity, nodule organogenesis, nodule senescence, ethylene signaling, hormones

## Abstract

Nodulation is the result of a beneficial interaction between legumes and rhizobia. It is a sophisticated process leading to nutrient exchange between the two types of symbionts. In this association, within a nodule, the rhizobia, using energy provided as photosynthates, fix atmospheric nitrogen and convert it to ammonium which is available to the plant. Nodulation is recognized as an essential process in nitrogen cycling and legume crops are known to enrich agricultural soils in nitrogenous compounds. Furthermore, as they are rich in nitrogen, legumes are considered important as staple foods for humans and fodder for animals. To tightly control this association and keep it mutualistic, the plant uses several means, including hormones. The hormone ethylene has been known as a negative regulator of nodulation for almost four decades. Since then, much progress has been made in the understanding of both the ethylene signaling pathway and the nodulation process. Here I have taken a large view, using recently obtained knowledge, to describe in some detail the major stages of the process. I have not only reviewed the steps most commonly covered (the common signaling transduction pathway, and the epidermal and cortical programs), but I have also looked into steps less understood (the pre-infection step with the plant defense response, the bacterial release and the formation of the symbiosome, and nodule functioning and senescence). After a succinct review of the ethylene signaling pathway, I have used the knowledge obtained from nodulation- and ethylene-related mutants to paint a more complete picture of the role played by the hormone in nodule organogenesis, functioning, and senescence. It transpires that ethylene is at the center of this effective symbiosis. It has not only been involved in most of the steps leading to a mature nodule, but it has also been implicated in host immunity and nodule senescence. It is likely responsible for the activation of other hormonal signaling pathways. I have completed the review by citing three studies which makes one wonder whether knowledge gained on nodulation in the last decades is ready to be transferred to agricultural fields.

## Introduction

Symbiotic nitrogen fixation is essential to agriculture. Graham and Vance (2003) estimated that about 50 million metric tons of atmospheric nitrogen was fixed by agriculturally relevant legumes annually. This nitrogen fuels much of the earth’s nitrogen cycle. Today, many farmers are moving to a more sustainable agriculture (as defined by Vance, 2001) as many of our soils are impoverished because of abuse. In an ideal world, we should bank more on symbiotic nitrogen fixation to remediate some of the detrimental effects our intensive agriculture has had on the environment. Vance (2001) outlined five recommendations toward which scientists have worked. Much has been done since: dozens of genes involved in the rhizobial symbiosis have been identified and mutants have been created to unravel both the roles played by these genes and the order in which they act. Now, an integrated approach must be taken to understand how the gene products fit together in a plant physiology context to make the mutualistic interaction as effective as possible.

Many reviews, varying in their approach and focus, have appeared recently on the roles played by plant hormones during nodulation (Desbrosses and Stougaard, 2011; Mukherjee and Ané, 2011; Murray, 2011; Ferguson and Mathesius, 2014). In general, all reviews underline that all known hormones are involved in the process as they tightly regulate every step from bacterial recognition to nodule senescence. Auxin, cytokinin, and ethylene, are thought to be essential actors and as such their roles have been studied in depth. To assign a specific role to any of these three hormones is nearly impossible because each hormone acts differently in space and time. However, it is generally accepted that cytokinin and auxin act positively (e.g., Mortier et al., 2014 and Mathesius, 2008, respectively), and ethylene negatively (Penmetsa et al., 2008), in the development of a nodule primordium (NP) and that cytokinin and ethylene have a negative effect on the progression of infection threads (ITs) (e.g., Murray et al., 2007 and Guinel and LaRue, 1992, respectively). Besides, to separate individual hormonal actions in a process such as nodulation is hardly possible because hormonal signaling pathways cross multiple times and in multiple places. For example, the three hormones cited above all have an effect on nodule positioning (Ferguson and Mathesius, 2014) and it is likely that the nodule position on the root is determined by an integration of their signaling pathways.

In this review, I have focussed on ethylene and its effect on nodule organogenesis, functioning, and senescence. Because of space constraints, I have restricted the review to the plant side of the mutualism, although I recognize this is a rather narrow view. I have assumed that legumes follow a similar blueprint in creating a nodule and in making it functional. However, this is likely incorrect since at least four different structural types of nodules are known (Guinel, 2009a). I have mentioned here only events occurring in indeterminate nodules, i.e., with a long-living meristem, and in determinate nodules, the meristem of which stops functioning early in the life-span of the nodule (Guinel, 2009a). I see this review as a foundation from which refinements can be made.

## A Review of Nodule Formation, Functioning, and Senescence

To describe effectively the effect ethylene has on nodulation, a review in some detail of the processes leading to a mature nodule is necessary. For reasons of space, I have simplified these processes as much as possible and I have mentioned only those genes which I thought could be involved directly or indirectly in an ethylene response. Readers interested in more in-depth reviews of nodule organogenesis *per se* or of specific steps in the process are invited to read Oldroyd and Downie (2008), Oldroyd et al. (2011), or Kondorosi et al. (2013).

### Pre-infection Events

**FIGURE 1:**
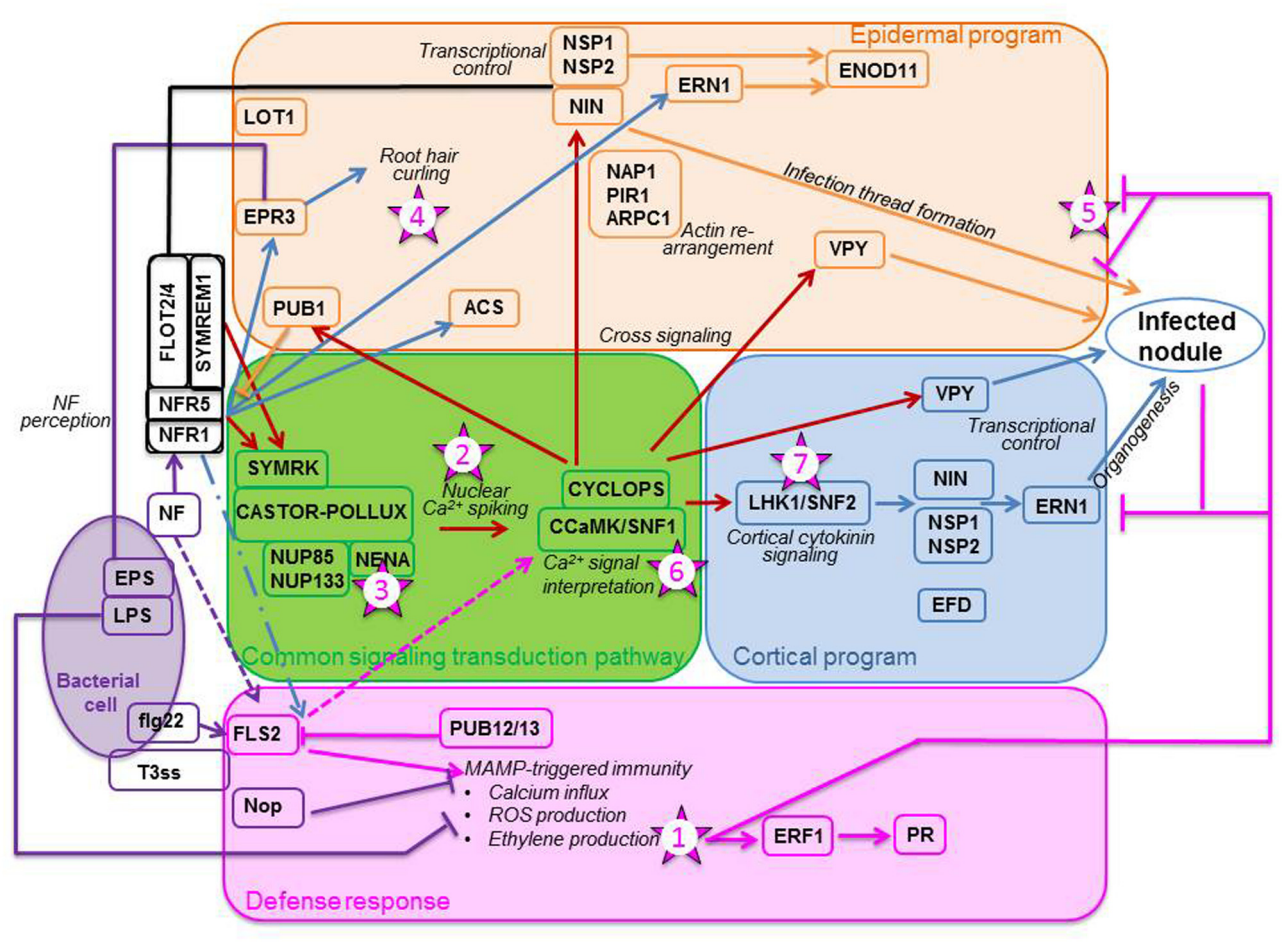
**Plant responses to the presence of rhizobia.** The bacterium (purple oval) triggers a defense response (pink box) by producing exopolysaccharides (EPS) and lipopolysaccharides (LPS), flagellin-like molecules (flg22), and type III-effector molecules (T3ss) used to inject Nop proteins in the plant cell. As the plant senses these molecules, especially flg22 with the FLS2 receptor, it mounts a set of defense responses. Among the outcomes are the production of ethylene and the up-regulation of pathogenesis-related (PR) proteins. Simultaneously, the rhizobium secretes Nod factors (NFs) which are perceived by the plant receptors NFR1 and NFR5, which may be recruited to membrane micro-domains by remorins (SYMREM1) and flotillins (FLOT2/4). Perception of NFs initiate the CSTP (green box) composed of eight genes: SYMRK, CASTOR/POLLUX, NUP85 and NUP133, NENA, CCaMK and CYCLOPS. CCAMK decrypts the calcium signal, triggering an epidermal program (orange box) and a cortical program (blue box). Epidermal program: Signaling, via CCaMK, triggers the ubiquitin ligase PUB1, considered a negative regulator of NFR1, and the transcription factor NIN which, with NSP1 and NSP2, and the vapyrin (VPY), affects the formation of the infection thread. For this event to occur, proteins important in the layout of the cytoskeleton, such as NAP1, PIR1, and ARPC1, are likely recruited. NF perception may also directly induce transcription of specific genes, such as the EPS receptor EPR3, the ethylene biosynthetic enzyme ACS, and an ethylene response factor required for nodulation ERN1. Cortical program: CCaMK triggers the cytokinin receptor LHK1 and the downstream transcriptions factors NIN, NSP1 and NSP2. In this program, in contrast to the epidermal program, ERN1 induction appears to be done through NIN and the NSPs. VPY and EFD, an ethylene response factor required for nodule differentiation, are also implicated in the program. The proper decoding of the calcium signal leads to nodule organogenesis. For a nodule to become infected and functioning, all steps must be impeccably orchestrated. Pointed arrows denote stimulation, flat arrows reflect inhibition, and broken arrows indicate speculative action or contradiction in the literature. The numbered stars represent potential location of ethylene signaling or action. The numbers correspond to the order in which these actions are reported in the text. Schematics adapted from Desbrosses and Stougaard (2011). Most of the genes mentioned on these diagrams are those which have been designated for *Lotus japonicus*; their orthologs for *Medicago truncatula* are mentioned in the text.

The rhizobium-legume interaction is initiated by the release of plant exudates such as flavonoids which attract rhizobia chemo-tactically toward the root. By binding to the rhizobial NodD1 protein, the flavonoids promote its affinity for the *nod* box (Peck et al., 2006), and thus initiate Nod Factor (NF) biosynthesis. NFs are recognized by the LysM receptor kinases Nod Factor Receptor1 (NFR1) and NFR5 (e.g., Desbrosses and Stougaard, 2011). Proper perception of NFs activates the common signaling transduction pathway (CSTP), the name of which alludes to the fact that this pathway is involved in the initiation of both rhizobial and arbuscular mycorrhizal symbioses (Kistner et al., 2005). In the symbiosis leading to nodulation, the CSTP (**Figure 1**, green box) initiates two distinct programs, the epidermal and the cortical programs of nodule organogenesis (Guinel and Geil, 2002). Recently, many reviews have been published on and around the CSTP (e.g., Desbrosses and Stougaard, 2011; Murray, 2011; Oldroyd, 2013).

Less often discussed in the pre-infection events are the defense responses that the legume must put in place when challenged with rhizobia. Recent studies have unraveled that a fine-balancing act is being played between the bacteria with their microbe-associated molecular patterns (MAMPs) production and the plant with its immune response elicitation (**Figure 1**; pink box) referred to as MAMP-triggered immunity (MTI; Gourion et al., 2015). Rhizobia produce not only NFs but also flagellin-like molecules (flg22) which are recognized by FLS2 (FLagellin-Sensing) receptors located in the epidermis plasmalemma (Khatabi and Schäfer, 2012). In response to these molecules, the host cell prompts a cascade of effects, such as calcium influx and production of reactive oxygen species (ROS). Furthermore, many genes such as those encoding peroxidases, chitinases, or ERFs (ethylene response factors) are up-regulated. This transient defense response is dependent on LjNFR1 (Nakagawa et al., 2011). Genes coding for Pathogenesis-Related (PR) proteins and a biosynthetic enzyme of the phytoalexin medicarpin are also up-regulated at the site of infection, the former transiently but the latter persistently (Breakspear et al., 2014). Bacteria have evolved to counteract these effects by secreting exopolysaccharides (EPS) and lipopolysaccharides (LPS); LPS inhibit ROS production whereas EPS are thought to chelate extracellular calcium ions preventing their cell entry (Gourion et al., 2015). To complement MTI, plants use another type of immunity known as effector-triggered immunity (ETI), which is set to respond to the direct injection of bacterial proteins, such as Nop proteins, in the cell cytoplasm via an effector (e.g., T3ss; Gourion et al., 2015). These bacterial proteins are known to inhibit MTI (**Figure 1**). The plant counter-attacks by encoding nucleotide-binding site/leucine-rich repeat proteins able to recognize the bacterial proteins (Gourion et al., 2015). Of interest for this review, hormones especially salicylic acid (SA), jasmonic acid (JA), and ethylene have been implicated in the setting of the response; these defense-related hormones likely cross-talk with DELLA proteins (Limpens et al., 2015) and hormones such as cytokinin and auxin (Zamioudis and Pieterse, 2012). Thus, there is an overlap between the defense and symbiotic pathways, with the defense reactions set up by the plant quickly suppressed (Gourion et al., 2015), allowing microbial entry and the potential successful rhizobial establishment in plant roots.

### Nodule Organogenesis

#### The Nod Factor Receptors

How the NFs mediate rhizobial entry whilst modulating defense is still not understood. As well, the mechanism behind the dual function of the NFRs to adjust to the dual action of the NFs, i.e., on the epidermal and cortical programs, has thus far not been uncovered, although some progress is being made (Limpens et al., 2015). Furthermore, the role played by each of the NFRs in activating the CSTP is still obscure. Mbengue et al. (2010) proposed that the entry receptor, of which LjNFR1 would be a component, sets off the epidermal program, whereas the signaling receptor, comprising LjNFR5, triggers the cortical program. How the two receptors work together to allow nodulation to proceed is difficult to envision. Mbengue et al. (2010) suggested that when NF binds to MtNFP, the ortholog of LjNFR5, PUB1 (Plant U-box protein), a U box-dependent E3 ubiquitin ligase, is phosphorylated by MtLYK3 (**Figure 1**), the ortholog of LjNFR1; this leads to its modulating the MtLYK3 downstream components by ubiquination. PUB1, considered a negative regulator of MtLYK3, is expressed early and transiently and its expression apparently requires the CSTP (Mbengue et al., 2010).

#### The Common Signal Transduction Pathway

The epidermal program encompasses all steps involving bacterial action, i.e., root hair (RH) curling, IT formation, and IT progression through the cortex, whereas the cortical program is responsible for the formation of the nodule infrastructure. For an efficient nodule to develop, both epidermal and cortical programs must not only be tightly regulated, but also accurately orchestrated (Guan et al., 2013). If the NFs are properly perceived and the Ca^2+^ signal correctly interpreted, then genes, the products of which regulate the two nodulation programs, **Figure 1**; Hayashi et al., 2010), are expressed downstream the CSTP. The correct expression of the genes comprised in the CSTP (**Figure 1**, green box) is required for nodulation success and if one of these genes is mutated, the nodulation process aborts (Oldroyd, 2013). It is possible for the two nodulation programs to be uncoupled since pseudo-nodules can form in the absence of bacteria; in such cases, the cortical program is activated on its own, independently of the epidermal program (for a review, see Guinel, 2009b). Such nodules form spontaneously on *Lotus japonicus* roots when the *CCaMK/SNF1* gene, coding for a calcium- and calmodulin-dependent kinase, is mutated (Gleason et al., 2006; Tirichine et al., 2006a) or when a phosphomimetic version of the *CYCLOPS* gene, coding for a phosphorylation substrate of CCaMK, is used (Singh et al., 2014). Evidence of the possible uncoupling of the two programs is also given by the mutant *Ljnena* (**Table 1**) which does not form ITs but exhibits nodules, albeit mostly empty (Groth et al., 2010). *NENA*, also of the CSTP (**Figure 1**), encodes a nucleoporin thought to work in concert with NUP85 as a scaffold protein within the nuclear pore complex (Groth et al., 2010).

#### The Epidermal Program

Much has been learned recently about the epidermal program (**Figure 1**; orange box). For example, within 24 hours of inoculation (hai), rhizobia induce the expression of remorin (MtSYMREM1 for SYMbiotic REMorin1; Lefebvre et al., 2010) and flotillins (MtFLOT2 and MtFLOT4; Haney and Long, 2010), two types of scaffolding proteins forming micro-domains in the plasmalemma. These proteins are thought to interact with MtLYK3 and MtNFP, maybe as a means to recruit them to their micro-domains (Lefebvre et al., 2010). MtSYMREM1 interacts with MtDMI2 (Does not Make Infections 2), an ortholog of LjSYMRK (SYMbiosis Receptor Kinase), located upstream of the CSTP CCaMK. *MtFLOT2* and *MtFLOT4* up-regulation requires the presence of nodule inception (NIN) and NSP2 (Haney and Long, 2010). Furthermore, MtFLOT4 is apparently required for proper IT elongation (Haney and Long, 2010). Recently, the plant receptor exopolysaccharide receptor 3 (EPR3) involved in epidermal bacterial entry was proposed to distinguish between EPS of compatible and incompatible rhizobia (Kawaharada et al., 2015). Its epidermal expression is triggered by NF perception and leads to RH curling (**Figure 1**). The importance of the actin cytoskeleton in the RH response is highlighted by three *L. japonicus* mutants, *Ljnap1* and *Ljpir1* (Nick-Associated Protein 1 and 121F-specific p53 Inducible RNA, respectively), and *Ljarpc1* (Actin-Related Protein Component 1) (Yokota et al., 2009; Hossain et al., 2012). NAP1, PIR1, and ARPC1 must play a role in the formation, maintenance and progression of the ITs because the mutants display aborted ITs in the epidermis and form non-colonized nodule primordia (Yokota et al., 2009; Hossain et al., 2012). An interesting mutant is *Ljlot1* (**Table 1**) since it is ethylene-insensitive. *Ljlot1* forms much less ITs than WT and thus displays few nodules; all are, however, functional (Ooki et al., 2005). The *Ljlot1* defect must be at the epidermal entry (**Figure 1**). In a recent RH transcriptomics study, Breakspear et al. (2014) showed that upon rhizobial infection the RH likely re-enters the cell cycle and that IT initiation is probably under auxin regulation.

**Table 1:** Characteristics of mutants displaying ethylene abnormality.

	*Ljnena-1*^∗^	*Ljlot1*	*Mtefd-1*	*Pssym16* (R50)	*Pssym15* (E151)
Mutation	Monogenic and recessive	Monogenic and recessive	Monogenic and recessive	Monogenic and recessive^1^	Monogenic and recessive^1^
		Unknown gene-product		Unknown gene-product	Unknown gene-product^2^
Nodule number	No infection threads Few pink nodules	Lower than WT by about 1/5th	Higher number of nodules	Significantly lower than WT^2^	Significantly lower than WT
Nodulation zone		No mention of it being atypical	Typical	Two, one close to the cotyledons, and one further down^3^	Two, one close to the cotyledons, and one further down^3^
Infection threads (IT)	Absent, infection resembles crack-entry	Normal in morphology but in low number	Numerous, especially in epidermis, and branched	Atypical, branched and convoluted^2^	Atypical, much branched and knobby^2^
Nodule primordia		All developed	Numerous and associated with ITs.	Consist of a single cell layer^2^	Consist of two cell layers^2^
Functional nodules	No	Yes	Broader and multi-lobed	Multi-lobed	Multi-lobed
			Defective in N_2_ fixation	With lower efficiency^4^	With lower efficiency^4^
Organ controlling nodulation phenotype		Root		Root^2^	Root^2^
Nitrate-sensitive		Higher sensitivity than WT	Similar to WT		Similar to WT^2^
Classical Triple response		Typical		Partial etiolation phenotype^5^	Typical^4^
Ethylene sensitivity	Ethylene required for nodulation to occur	Insensitivity in terms of nodulation	Expression independent of ethylene	Roots sensitive as in WT^5^ Shoots insensitive to ethylene^5^	Sensitive to silver^2^ Insensitive to ACC or AVG^2^
Ethylene evolution				As WT^4^	
Cytokinin sensitivity			Likely targets the cytokinin response regulator MtRR4	Accumulator of cytokinin because of a defective cytokinin oxidase enzyme^6^	Accumulator of cytokinin^2^
ABA sensitivity					Hyper-sensitive to ABA when in the vegetative stage^2^
Root morphology of non-inoculated plants		Shorter roots			PR length similar to WT^2^ Young: Fewer LR^2^ Old: Many more than WT^3^
Other traits		Moderately dwarf Wavy trichomes on the calyx and abaxial side of leaflets		Short and thick epicotyl^5^ Pale green leaves^2^	Shorter plants in nitrogen-limited conditions^4^
Response to mycorrhizal fungi	Low AM frequency as infection aborts after epidermal invasion	Typical, as in WT	Typical, as in WT	Typical, as in WT^4^	Hyper-mycorrhizal^2^
References	Groth et al., 2010	Ooki et al., 2005	Vernié et al., 2008	^1^Kneen et al., 1994	^1^Kneen et al., 1994
	^∗^Temperature-dependent			^2^Guinel and Sloetjes, 2000	^2^Jones et al., 2015
				^3^Remmler et al., 2014	^3^Remmler et al., 2014
				^4^Unpublished data	^4^Unpublished data
				^5^Ferguson et al., 2005	
				^6^Held et al., 2008	


#### The Cortical Program

Nodule organogenesis requires the dedifferentiation of the nodule progenitor cells, which are likely the target of the NF signal in the cortex. Once these cells have re-acquired the capability of dividing, they organize to form a NP and a nodule meristem (NM). As the NM grows outward toward the root surface, the IT grows inward toward the NP (Guinel and Geil, 2002) under the guidance of pre-ITs (for more details, see Murray, 2011). Nodule development necessitates the expression and regulation of many genes (**Figure 1**; blue box) and the involvement of many hormones. Cytokinin for example is known to play an essential role in nodule formation as *L. japonicus snf2* plants, which have a gain-of-function mutation in the *LHK1* cytokinin receptor gene, produce spontaneous nodules independently of CCaMK (Tirichine et al., 2007). That NF-induced cell reprogramming is dependent on a functional receptor was confirmed by van Zeijl et al. (2015) in a *Medicago truncatula* transcriptomics study; using a synthetic cytokinin reporter gene, these authors localized the cytokinin response in the cells known to be involved in NP formation. *LjLHK1* expression in the cortical cells increases as the NP enlarges until the nodule reaches the point of emergence (Held et al., 2014). A mutant of interest is *Mtefd-1*; it has its place in the cortical program because the expression of ERF required for nodule differentiation (*EFD*), in the central region of the nodule, can be triggered by a defective bacterial mutant (Vernié et al., 2008). As the *Mtefd-1* mutant forms many ITs and numerous NP which do not proceed correctly to maturity (Vernié et al., 2008), it is likely that *EFD-1* (**Table 1**) known to activate the expression of a cytokinin response regulator is a negative player in nodule initiation and is required for the late stages of nodule development.

#### Coordination of the Two Programs

Few genes located downstream of *CCaMK* are known to be involved in both programs. First, the transcription factor (TF) NIN is thought to affect negatively the rhizobial infection but positively the cortical program (Yoro et al., 2014). Second, NSP2 and NSP1, two GRAS domain TFs thought to be indispensable in bridging CCaMK to downstream actors (Heckmann et al., 2006), act in a coordinated manner since their mutant phenotypes are similar. Despite RH deformation and calcium spiking, neither ITs nor NP form in these mutants (Heckmann et al., 2006). Third, the protein vapyrin (VPY), containing a Major Sperm Protein domain and a series of ankyrin repeats, is thought to be implicated in membrane biogenesis and trafficking because of its subcellular localization (Murray et al., 2011). In *vpy* mutants, there are more ITs and NP than in WT; however, the epidermis-arrested ITs are misshapen and the NP are not infected (Murray et al., 2011). Other mutants which should be placed in this group are *Mtbit1-1* and *Mtbit1-2*, now known as *MtERN1* (ERF Required for Nodulation). Both mutants display ITs not progressing beyond the epidermis and delayed NP not maturing (**Figure 1**; Middleton et al., 2007). In WT, *MtERN1* is not only expressed early in the nodule progenitor cells but also later in the cortical cells surrounding the IT (Cerri et al., 2012). As NP development is arrested in the mutant and *MtERN* expression requires *MtCRE1* (Plet et al., 2011), an ortholog of *LjLHK1*, Cerri et al. (2012) suggested that ERN1 is required for late nodule development. It is possible that the products of these TFs have different roles in the two nodulation programs, as for NIN.

When the cortical program is completed, the nodule structure is well organized. Depending on the types of legumes bearing them, the nodules are spherical (determinate) as on *L. japonicus* and soybean roots or oblong (indeterminate) as on *M. truncatula* or pea (*Pisum sativum*) roots (Guinel, 2009a). The former quickly loses NM activity whereas the latter with an active NM displays a characteristic zonation with six zones: the meristematic zone (zone I), the infection zone (zone II), the interzone II–III, the fixation zone (zone III), the senescing zone (zone IV) and the saprophytic zone (Zone V; see Guinel, 2009a for more details). In this indeterminate-type nodule, a specific cell borne in the meristematic zone passes through all the nodule zones and becomes infected or non-infected (Kondorosi et al., 2013). Recently, Xiao et al. (2014) demonstrated that not all nodule cells have a similar origin, whereas those located deep in the nodule originate from the NP, those at its distal end originate from the NM.

Via the dual action of several genes in both programs and a constant communication between the symbionts, likely through NF perception, nodule organogenesis proceeds according to a planned choreography. One of the controls relies on the constant perception and turnover of NFs within the developing and mature nodule. Thus, recently, Moling et al. (2014) localized the two NF receptors, MtLYK3 and MtNFP, to the cell periphery of two cellular layers located at the boundary between zones I and II. These receptors are also localized within the cell vacuoles. Moling et al. (2014) proposed that the periphery-located receptors, in addition to regulating the bacterial release in these cells, are involved in dampening plant defense responses whereas those receptors located in the vacuoles are targeted for degradation.

### Internal Colonization of the Bacteria

#### Bacterial Release in the Infection Zone

**FIGURE 2:**
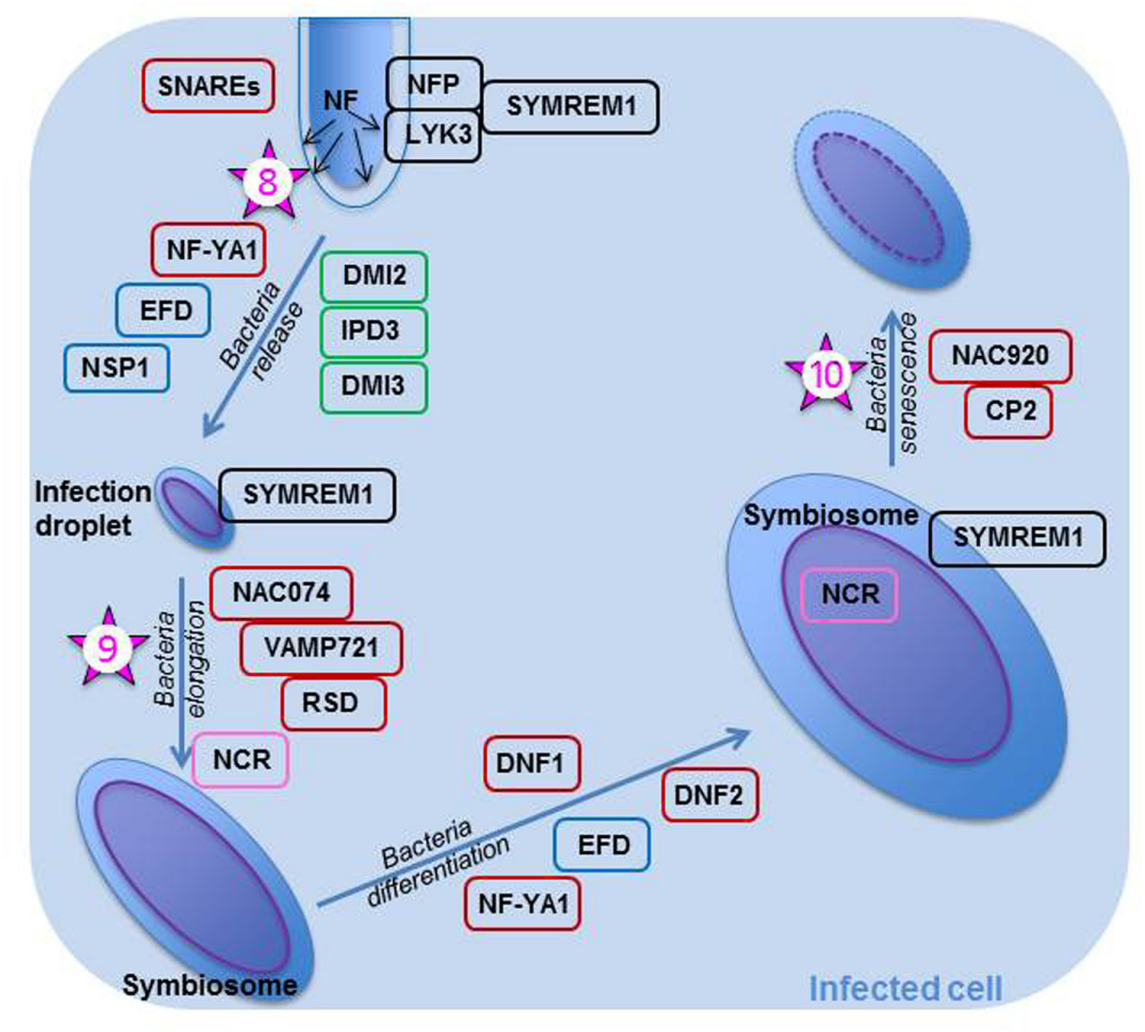
**Rhizobial release and bacteroid differentiation within an infected plant cell of *Medicago truncatula*.** Once the rhizobia are released in the infection droplets, they divide and elongate. They later on differentiate into bacteroids; many bacterial genes, such as those necessary for division, are then turned off, whereas genes the products of which are necessary for bacteroid metabolism (nitrogenase, transporters, etc…) are turned on. As all these events are important steps in the symbiosis, they are controlled tightly and many genes and their products are implicated in their control. All proteins within a black box are known to be involved with the NF perception. Those in a green box are known to be part of the common symbiotic transduction pathway. Proteins in a blue box are thought to play a role in both the epidermal and cortical nodulation programs. Proteins in a brown box are not known to play a role early in the nodulation process; they may be specific for controlling these specific steps. NFs are continuously produced by rhizobia within the infection thread and when the rhizobia are released, the NFs are perceived by NFP and LYK3, the Nod factor receptors. These receptors are likely to be recruited in a micro-domain by SYMREM1 expressed in the plasmalemma of the infected cell. SNAREs and NF-YA1 are essential in bacterial release, whereas NAC074, VAMP721, and RSD are required for bacterial elongation. The bacteria differentiate once they have perceived the antimicrobial NCRs, which are found in the bacteroid after they crossed both symbiosome and bacterial membranes. DNF1 is necessary for the entry of NCRs in the symbiosome. Bacterial differentiation is also under the control of DNF2. Finally, MtNAC920 working in concert with CP2 trigger bacteroid senescence. The numbered stars represent potential location of ethylene signaling or action and the numbers correspond to the order in which these actions are reported in the text.

If all the steps of early nodule development are performed correctly and the IT progresses without any mishap through the cortex, then the IT reaches the infection zone of the young nodule where it delivers un-walled infection droplets (**Figure 2**) in cells which are polyploid (Mergaert et al., 2006). Intracellular bacterial accommodation is linked to both high secretory activity and intense vesicle trafficking in the newly infected cells, as large amounts of endo-membranes are produced (Bapaume and Reinhardt, 2012). Bacteria surrounded by the peribacteroid membrane make up an organelle-like structure known as a symbiosome. The bacterial delivery is performed under tight genetic control (**Figure 2**) as demonstrated by the work of Ivanov et al. (2012) who prevented bacterial release into the cytoplasm of the infected cell by silencing two genes coding for soluble *N*-ethylmaleimide sensitive factor attachment receptor (SNARE) proteins. Scaffolding proteins such as MtSYMREM1 and FLOT2/FLOT4 are also necessary for proper bacterial delivery (Lefebvre et al., 2010, and Haney and Long, 2010, respectively). MtSYMREM1 has been located at the plasmalemma lining the IT and onto the infection droplets in zone II, as well as to the symbiosome membrane in zone III (Lefebvre et al., 2010); as was mentioned earlier, the protein may act in recruiting the NF receptors into a microdomain. The two NF receptors are also important in rhizobial release (**Figure 2**). They are localized on the cell membrane lining the IT and removed from that membrane when the bacteria are released in infection droplets (Moling et al., 2014). MtIPD3 (Interacting Protein of DMI3), ortholog to LjCYCLOPS and known to interact with CCaMK (Ovchinnikova et al., 2011), NF-YA1 previously known as HAP2, a transcription regulator known to control both IT progression through the cortex (Laporte et al., 2014) and NM development in indeterminate nodules (Combier et al., 2006; Xiao et al., 2014), and MtEFD (Vernié et al., 2008), are all involved in bacterial release (**Figure 2**), because mutations in any of these proteins prevent the rhizobia from leaving the IT. In the bacterial discharge from ITs, MtIPD3 was shown to interact with MtDMI2 and MtDMI3 (Ovchinnikova et al., 2011). NSP1 has also been suggested to play a role in proper bacterial release (Heckmann et al., 2006).

This crucial step is subjected to the “scrutiny” of the plant which uses its defense system to assess whether or not the infecting rhizobia are welcome, as discussed by Lang and Long (2015) in their transcriptomics study of nitrogen fixation-defective mutants of *M. truncatula*. Thus, in nodules arrested early in development, one finds a high abundance of transcripts for ascorbate, glutathione, and proteins involved in ROS detoxification; these proteins if expressed would increase resistance against an inappropriate level of biotic stress (Lang and Long, 2015). Recently, in a study designed to better understand nitrogen-induced senescence, Karmarkar (2014) found that nodules treated with inhibitory NH_4_NO_3_ concentrations specifically express *MtNAC074*, a TF that binds directly to the promoters of *MtVAMP721* genes, which are coding for members of the vesicle associated membrane protein (VAMP) family. Nodules over-expressing *NAC074* had symbiosomes delayed in development with an atypical cell arrangement and a lower nitrogenase activity than controls. Karmarkar (2014) proposed that MtNAC074 negatively affects symbiosome development because it represses VAMP721s which regulate many processes in plants, including delivery of cargo essential to symbiosome formation. Of interest is the recent characterization of *MtRDS* (Regulator of Symbiosome differentiation), a mutant exhibiting early senescence with incorrectly differentiated bacteroids (Sinharoy et al., 2013). *MtRSD* is expressed in zone II and interzone II–III; it codes for a TF belonging to the C_2_H_2_ family. RSD binds directly to the *VAMP721a* promoter and in doing so it represses VAMP721a production via its EAR domain (Sinharoy et al., 2013). EAR-repressors have been implicated in the suppression of defense and stress genes (Kazan, 2006) and may thus lower the plant defense responses during rhizobial release in the invasion zone.

It is becoming apparent that many genes involved in the initial stages of the nodulation process are also implicated in the release of rhizobia (Moreau et al., 2011). This may not be surprising knowing that NFs are being continuously produced and are active from the time of inoculation to that of rhizobial release into the infected cell (Gourion et al., 2015). However, later during bacterial differentiation, when NFs are likely no longer synthesized, different plant genes come into play, suggesting different control strategies (Lang and Long, 2015). In views of Xiao et al.’s (2014) study, it is also likely that rhizobial release in NP cells is controlled differently from that in NM daughter cells because NF-YA1 is essential for bacterial release in the latter but not in the former.

#### Bacterial Symbiosome

The symbiosome is an enclosed space where the rhizobium, unable yet to fix nitrogen, differentiates into a bacteroid capable of nitrogen fixation. Bacteroid differentiation depends on the plant host; thus, the events occurring will differ in determinate and indeterminate nodules (Mergaert et al., 2006; Kondorosi et al., 2013). In plants such as *Medicago* or *Pisum*, which form indeterminate nodules, the trigger for bacteroid differentiation is the production by the infected cell of antimicrobial nodule-specific cysteine-rich (NCRs) which are targeted via a specific signal peptide to the symbiosome (**Figure 2**; Van de Velde et al., 2010). NCR proteins cross the symbiosome membrane and enter the bacterial cytosol where they modulate bacteroid maturation (Van de Velde et al., 2010). NCR peptides involvement in controlling bacterial release during the intermediate and late symbiotic stages was confirmed by the transcriptomics study of Lang and Long (2015). For these NCRs to be synthesized and sent to their target, the Mt*DNF1* (Defective in Nitrogen Fixation) gene encoding a subunit of a signal peptidase complex must be properly expressed in zone II (Wang et al., 2010). Additionally, *MtDNF2* which encodes a predicted phosphatidylinositol phospholipase C-like protein must be essential for bacteroid differentiation and maintenance, because its mutant exhibits early bacteroid senescence in zone III (Bourcy et al., 2013). Proteins important in bacterial release also play a role in bacteroid differentiation; thus, *MtNF-YA1* and *MtEFD* are implicated in this step, since their mutants display nodules with bacteria arrested in development (Combier et al., 2006 and Vernié et al., 2008, respectively).

### Nodule Functioning in the Fixation Zone

As the nodule grows in size, the infected cells progress through the interzone II–III; it is in this zone that bacteroids differentiate and leghaemoglobin is synthesized (de Billy et al., 1991). The cells in this zone are metabolically active and contain many organelles, with the infected cells depending much on the metabolism of the non-infected cells. In the infected cells, the bacteroids fix nitrogen once their nitrogenase enzyme complex is turned on. The symbiosome has now become a compartment where a large amount of nutrient exchange occurs (Bapaume and Reinhardt, 2012; Clarke et al., 2014). Whereas ammonia, the product of nitrogenase, exits the symbiosome through ammonium transporters, carbohydrates required to fuel nitrogen fixation enter this space as dicarboxylic acids (Lodwig et al., 2003) via dicarboxylate transporters (e.g., Udvardi et al., 1988). For nitrogen fixation to occur, the bacteroids must be provided with a low amount of branched amino-acids (Prell et al., 2009), which are transported across the symbiosome compartment via ABC transporters (Prell et al., 2010). In some rhizobia-legume associations, the bacteroids in effect have become auxotrophic for these amino-acids, highlighting their metabolic dependence on the infected cell (Prell et al., 2009, 2010). The symbiosis has evolved in such a way that the rhizobial and plant requirements are all being met. As examples, to protect the bacterial nitrogenase enzyme from too high levels of oxygen, the plant hosts the bacteria in the center of the nodule, and to transport photosynthates to the nodule and nitrogeneous compounds to plant sinks, nodule vasculature develops in the nodule cortex (Guinel, 2009a,b). Nitrogen fixation is required for the bacteroids inside the nodule to remain alive; once nitrogen stops being fixed, a defense-like mechanism kicks in to degrade the ineffective bacteroids. According to Berrabah et al. (2015), each step involved from rhizobial release to bacteroid death is controlled but likely not in the same way.

### Nodule Senescence

Nodule senescence is under tight control, genetic as well as hormonal. The events described below are likely linked to the cell-cycle arrest in the NM and to the cessation of bacterial release from the IT in the infection zone. In natural conditions, one of the first visible signs of nodule senescence is a shift of nodule color from pink to green, because plant leghemoglobin is no longer expressed once the nitrogenase activity stops (Puppo et al., 2005). In an indeterminate nodule, the steps making up the process are well orchestrated (Guerra et al., 2010). The same is likely true in a determinate nodule, but the means by which the control is taking place may be dissimilar temporally and spatially (Puppo et al., 2005). Also there may be differences in metabolism because indeterminate senescing nodules of pea have their superoxide and H_2_O_2_ levels decline while these levels increase in the determinate senescing nodules of soybean (Puppo et al., 2005). However, both nodule types go through the same physiological changes as they move from being a sink to become a source of nutrients for the plant (Guerra et al., 2010).

In *M. truncatula*, the first signs appear in the proximal area of zone III, and the event spreads in such a manner that the senescent tissue takes a cone-shape (Guerra et al., 2010). Bacteroids are first to degrade within the symbiosomes; then, once the plant cells have resorbed the symbiosome content, they start to deteriorate (Van de Velde et al., 2006). The infected cells collapse first while the non-infected cells mine the remobilized nutrients (Van de Velde et al., 2006) which are directed to the nodule vasculature. Cysteine proteases, likely active in mobilizing the nutrients from the degrading symbiosomes (Guerra et al., 2010), are the earliest molecular markers of the process (Van de Velde et al., 2006). Two genes, the expression of which is also up-regulated earlier, code for members of the AP2/ERF family usually expressed during stress responses and host immunity (Van de Velde et al., 2006). Later on, when the process is more advanced, genes coding for proteases, a vacuolar processing enzyme (VPE)-precursor, proteasome complexes, and catabolic enzymes are all up-regulated (Van de Velde et al., 2006). The expression of these genes reflects the high metabolic activity required for the recycling of the components of the symbiosomes and infected cells. Karmarkar (2014) in his nitrate-induced senescence study demonstrated the importance of the TF MtNAC920 in the process (**Figure 2**). *MtNAC920* triggers nodule senescence by directly targeting the promoter region of *MtCP2*, a gene coding for a cysteine protease.

As for hormones, abscisic acid (ABA), ethylene, and JA have all been shown to be involved (Puppo et al., 2005; Hichri et al., 2015). Puppo et al. (2005) proposed a model whereby ABA plays a primary role in coordinating senescence. According to these authors, as the nodule ages, the ascorbate levels decrease and the carbon/nitrogen ratio increases in the nodular tissues; this results in ABA synthesis and its transduction pathway activation, leading to an increase in proteases and proteasome activities. The importance of ABA was not confirmed by the *Medicago* transcriptomic study of Van de Velde et al. (2006), who found gene tags suggesting the involvement of three other hormones. For these authors, ethylene and JA act positively during senescence while gibberellic acid (GA) acts negatively. JA positive action is suggested by the up-regulation of several lipoxygenase genes whereas GA negative role is indicated by the up-regulation of the gene coding for GA2-oxidase, an enzyme known to inactivate GA (Van de Velde et al., 2006). Recently, new evidence was provided toward ethylene having an active role in senescence, at least in stress- and nitrate-induced nodule senescence (Karmarkar, 2014); this will be elaborated upon further below.

## A Review of Ethylene Biosynthesis and Signaling

### Ethylene Biosynthesis

**FIGURE 3:**
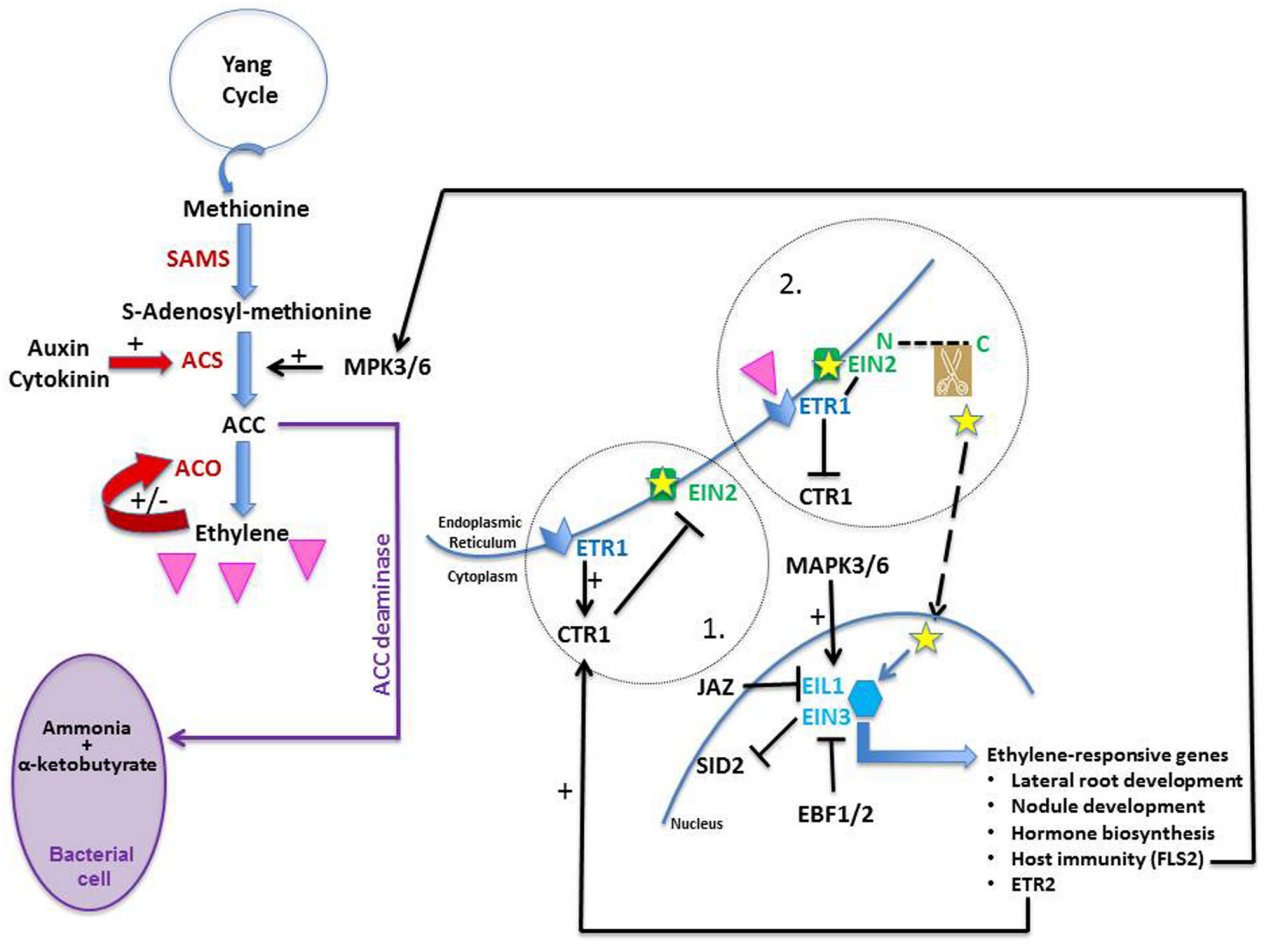
**Ethylene biosynthesis and transduction pathway.** Methionine is the precursor of ethylene (pink triangle). It is converted into *S*-adenosyl-methionine (SAM) by SAM synthetase (SAMS); SAM is converted into 1-aminocyclopropane-1-carboxylate (ACC) by ACC synthase (ACS). ACC is later converted into ethylene by ACC oxidase (ACO). Both auxin and cytokinin are known to promote ethylene production; the former up-regulates ACS expression whereas the latter prevents ACS degradation. Ethylene is known to modulate its own levels by stimulating (+) or inhibiting (-) ACO activity. ACC deaminase is an enzyme found in certain types of bacteria; it converts ACC into ammonia and α-ketobutyrate and both may be used as nutrients by the bacteria. When no ethylene is sensed in a cell (circle 1), ethylene receptors (such as ETR1) located on the ER membrane promote CTR1 activity; in doing so, they allow CTR1 to phosphorylate EIN2 which inactivates it. Upon ethylene perception (circle 2), ETR1 is dephosphorylated and CTR1 is switched off. ETR1 can now bind efficiently to EIN2 which is activated. Its C-end terminus (small yellow star) is cleaved and moves to the nucleus where it stabilizes the transcription factors EIN3 and EIL1, which are then able as dimers to trigger the expression of ethylene-responsive genes, inducing many developmental and biochemical events. When ethylene is not perceived, EIN3 and EIL1 are targeted to proteasomes via the action of EBF1/2. MAP kinases 3 and 6 (MAPK3/6) are known to activate ACS enzymes and to phosphorylate EIN3 in *Arabidopsis*, protecting it from degradation. Jasmonic acid is known to work synergistically with ethylene by promoting the degradation of JAZ2, a protein known to repress EIN3/EIL1. Finally, EIN3/EIL1 inhibits salicylic acid synthesis as they bind to the promoter of SID2, one of its biosynthetic genes, thus inactivating it.

The ethylene biosynthesis pathway is relatively simple and a good review on the subject was published by Lin et al. (2009). In short, there are three steps in the pathway (**Figure 3**). Methionine, its precursor, is provided by the Yang cycle which is one of the mechanisms used by plants to recycle sulfur. With the addition of an ATP molecule, methionine is converted into *S*-adenosyl methionine (SAM) by the enzyme SAM synthetase (SAMS). The rate-limiting step which commits SAM to ethylene synthesis is regulated by ACS (ACC synthase) which catalyzes the conversion of SAM into ACC (1-aminocyclopropane-1-carboxylate). In the presence of oxygen and ascorbate, ACC is converted into ethylene, cyanide, and CO_2_ via the action of ACO (ACC oxidase). ACC can also be converted via ACC deaminase into ammonia and α-ketobutyrate (**Figure 3**). Bacteria as well as plants are known to possess that enzyme (Van de Poel and Van Der Straeten, 2014); the former may use it to decrease the ethylene levels of the latter (e.g., Klee et al., 1991; Gamalero and Glick, 2015).

ACS and ACO are both encoded by multigene families. The control of ethylene biosynthesis is thought to rest mostly on the regulation of the ACS isozymes. However, it is more than likely that control occurs too via ACO because (1) more than one ACO isozyme exist and these exhibit different temporal expressions (e.g., Jafari et al., 2013); (2) endogenous ethylene promotes its own synthesis during pea germination by stimulating ACO activity (**Figure 3**; Petruzzelli et al., 2000); (3) a feedback mechanism with ethylene inhibiting ACO has been suggested based on ACO transcript levels in *M. truncatula* inoculated by rhizobia (Prayitno et al., 2006a); and (4) Larrainzar et al. (2015) found in *M. truncatula* that the expression of one ACO is inhibited by ethylene whereas three ACOs require proper ethylene signaling to be induced.

ACS is under tight regulation, mainly via the control of its degradation rate (Rodrigues et al., 2014). In *Arabidopsis thaliana*, the stability of each ACS member depends on a specific domain located in the C-terminal end. Based on this domain, one can recognize three ACS classes (Types I–III) and each class exhibits distinct regulatory features (Rodrigues et al., 2014). Type II class-ACS members have a short C-terminus domain containing a putative calcium-dependent protein kinase target site (Rodrigues et al., 2014), which if altered prevents the degradation of the ACS enzyme as it is no longer targeted to the proteolytic machinery (Chae et al., 2003). This is seen in *Ateto2* and *Ateto3*, two mutants with mutations in the ACS5 C-terminal domain. Both mutants are ethylene over-producers, not because the mutation is stimulating the enzyme but rather because its degradation is prevented (Chae et al., 2003). An interesting mutant is *Ateto1*, also an overproducer of ethylene, but for a different reason. *Ateto1* is not mutated in ACS5 but in a protein involved in the proteasome-dependent degradation pathway. Upon binding of ETO1 to the ACS5 C-terminal domain, ACS5 is targeted for degradation (Yoshida et al., 2006). In *Ateto1*, because ETO1 is dysfunctional, ACS5 is stable and ethylene is overproduced (Chae et al., 2003).

It is worthwhile here to note that ethylene production, at least in *Arabidopsis*, is also under hormonal control. For example, cytokinin and auxin, two hormones known to play a role in nodulation, have been shown to promote its synthesis. The former promotes ethylene production by reducing the turnover rate of ACS5 (Chae et al., 2003; Hansen et al., 2009). The latter induces ethylene biosynthesis by promoting, in a cell-specific manner, all *ACS* expression, except for *ACS1* (Tsuchisaka and Theologis, 2004).

### Ethylene Signaling

Recently, great progress has been made in this field as can be seen in many excellent reviews (e.g., for ethylene receptors, Lacey and Binder, 2014; and ethylene signaling, Merchante et al., 2013). In a nut-shell, ethylene is perceived by several receptor proteins, e.g., ETR1 (**Figure 3**, 1) or ETR2, which are located on the ER membrane (Chen et al., 2002); the receptors are negative regulators of signaling. Upon being triggered, the receptors are inactivated. This has two effects: (1) their de-phosphorylation which allows them to bind more efficiently to Ethylene INsensitive 2 (EIN2; Cho and Yoo, 2015), a protein also localized on the ER membrane (Bisson et al., 2009); and (2) the switching off of the protein Constitutive Triple Response 1 (CTR1) which is no longer capable of phosphorylating EIN2 (**Figure 3**, 2). Once removed from the CTR1 influence, EIN2 is subjected to a structural modification as its C-terminus is cleaved (**Figure 3**, 2). This fragment is moved physically to the nucleus where it stabilizes the TFs ethylene insensitive 3 (EIN3) and EIL1 (EIN3-Like 1), both responsible for the regulation of ethylene-responsive genes (Ju et al., 2012); it does so likely by degrading EBF1/2, F-box proteins which would otherwise target EIN3 and EIL1 to proteasomes. Functioning as dimers, EIN3 and EIL1 trigger the expression of multiple ethylene-regulated genes, among which one can find the *ETR2* gene. Its gene-product, the ethylene receptor ETR2, activates the negative regulator CTR1 which as a result phosphorylates EIN2 which deactivates it. In this manner, ethylene signaling can be tuned down in the absence of additional ethylene (Merchante et al., 2013). Finally, some downstream targets of EIN3 are essential components of other hormonal signaling pathways, illustrating the intricacy of plant development regulation. Although understanding how EIN2 and CTR1 interact has filled a physical gap between the ER and the nucleus, there is still much to learn about ethylene signaling; for a recent review pointing to knowledge gaps in the field of ethylene signaling, see Cho and Yoo (2015). There may be yet surprises in the pathway as new CTR1-independent signaling routes are being proposed (Zhang et al., 2014).

## Nodulation and Ethylene

Ethylene has many roles in the development of a plant, from seed dormancy to fruit ripening (Abeles et al., 1992). In nodulation, its effect was reported as early as four decades ago when rhizobia-inoculated root cultures of bean were treated with ethylene; as a result, not only nodule numbers decreased dramatically but the amount of nitrogen fixed was also reduced (Grobbelaar et al., 1971). Forty years later, we have a better understanding, although still far from being complete, of the effects the hormone has on the nodulation process. In the last decade or so, we have learned much from ethylene signaling pathway mutants and from transgenic plants carrying ethylene-related genes.

### EIN2 in Model Legumes

The mutant *sickle* from *M. truncatula* proved to be especially useful; it has indeed been a determinant in getting a better grip on the role of ethylene in nodulation. *Mtskl*, first characterized by Penmetsa and Cook (1997) as a hyper-nodulation mutant, forms 10–30 times more nodules than the wild-type (WT) plants (**Table 2**). Because on younger plants the nodules it forms are all located in the typical nodulation zone, i.e., the most susceptible nodulation zone (Bhuvaneswari et al., 1981), they are tightly pressed one against the other. The gene *Mtskl* affects IT resilience; thus most ITs are associated with NP which develop, albeit with a delay, into nodules capable of typical nitrogen fixation (Penmetsa and Cook, 1997). The mutant is insensitive to ethylene as, for example, it failed to exhibit the classical “triple response” when treated with ACC or ethylene (Penmetsa and Cook, 1997). *Mtskl* ethylene-insensitivity was confirmed by Penmetsa et al. (2008) who demonstrated that MtSKL is an ortholog of AtEIN2. It is an integral membrane protein which comprises an N-terminal sequence similar to that seen in proteins belonging to the natural resistance-associated macrophage protein (NRAMP) family and a unique C-terminal sequence. Its hydrophobic core composed of 10 trans-membrane domains is located within its N-terminal end (Penmetsa et al., 2008). To date, a single EIN2 gene has been found in *M. truncatula* (Penmetsa et al., 2008) and in pea and chickpea (Weller et al., 2015), whereas two have been identified in common bean (Weller et al., 2015), in soybean (Miyata et al., 2013), and in *L. japonicus* (Chan et al., 2013). The two *L. japonicus* genes *LjEIN2a* and *LjEIN2b* are expressed in all organs examined, including roots and nodules (Miyata et al., 2013).

**Table 2:** Characteristics of the mutants and transgenic plants altered in the protein EIN2.

	*Mtskl*	*Ljenigma-1*	*LjEIN2* (1 and 2)	*etr1-1*	*ERS1*
Mutation	Single recessive mutation^1^ of MtEIN2^2^	LjEIN2-2 (also named LjEIN2a) Recessive mutation	RNAi constructs targeting both genes	Transgenic plants containing a vector with *Atetr1-1*^∗^	Transgenic plants with a vector carrying a mutated *CmERS1*^∗^
Nodule number	10–30 × more ^1^ ^and 3^	3 × less	Together 3 × increase	Larger nodule number Smaller nodules	Higher number of nodule primordia but similar nodule number
Nodulation zone	Typical^1^		Clustered in a limited region	Typical	Typical
Infection threads	Persistent^1^	Typical and fewer		Increased number	Significantly higher
Nodule positioning	Atypical^1^	Atypical		Atypical	
Organ controlling nodulation phenotype	Root^6^	Root and shoot			
Nitrate-sensitive	Yes^4^	Increased sensitivity to high nitrate levels		Yes	
Classical Triple response	Lack of^2^	Lack of		Lack of	
Cytokinin sensitivity	Decreased^2^				
ABA sensitivity		Increased sensitivity Higher ABA production			
Root morphology of non-inoculated plants	Longer primary root^4^ Delayed LR growth^4^ Thin^5^ Longer cortical cells^5^ Shorter RH^3^	Increased root elongation Lightly increased LR number Greater root DW	Longer roots Shorter RH	Smaller LR number	
Other traits	Delayed leaf senescence^1^ Delayed petal senescence^1^ Decreased abscission of seed pod and leaves^1^	Slower plant growth Delayed flowering time Smaller seed pods Smaller leaves		Delayed flowering time Delayed petal senescence Delayed ripening of fruit	Delayed petal senescence
Ethylene sensitivity Ethylene evolution	Ethylene insensitive^1^ Absence of an ethylene-mediated ethylene production^2^	Seedling root levels five times as high as those of WT		Ethylene-insensitive	Ethylene-sensitive as seen by macro-observations and growth of ACC-treated plants
Response to mycorrhizal fungi	Increased^2^	As in WT			
Response to pathogens	More susceptible^2^				
References	^1^Penmetsa and Cook, 1997	Chan et al., 2013	Miyata et al., 2013	Lohar et al., 2009	Nukui et al., 2004
	^2^Penmetsa et al., 2008			^∗^Constructs with a gene from *Arabidopsis thaliana*	^∗^Constructs with a gene from *Cucumis melo*
	^3^Oldroyd et al., 2001				
	^4^Prayitno, 2010				
	^5^Prayitno and Mathesius, 2010				
	^6^Prayitno et al., 2006b				


The presence of two *EIN2* genes in common bean, soybean and *L. japonicus*, all plants forming determinate nodules, may explain partly the conundrum exposed about 15 years ago when it was shown that in soybean nodules formed in the presence of ethylene (Lee and LaRue, 1992a) and on ethylene-insensitive mutants (Schmidt et al., 1999). These observations led to the hypothesis that the formation of indeterminate and determinate nodules may be regulated differently by ethylene. Yet, some legumes forming determinate nodules are known to respond to ethylene. For example, beans treated with ethephon (a compound which spontaneously releases ethylene) and *Macroptilium atropurpureum* treated with ACC exhibit a smaller number of nodules than non-treated plants, and either plant treated with amino-vinyl-glycine (AVG), an inhibitor of ACS, forms a larger number of nodules (Nukui et al., 2000; Tamimi and Timko, 2003). Furthermore, ethylene sensitivity, assessed by leaf senescence and chitinase activity assays, was shown to depend on soybean cultivars (Xie et al., 1996). These results led Nukui et al. (2000) to propose that recent breeding processes may have selected for soybean lines which were ethylene-insensitive. A differential in ethylene response is also seen in ethylene-insensitive *Lotus* transgenic plants carrying a vector with the mutated *etr1-1* from *Arabidopsis* (Lohar et al., 2009). Different lines classified according to their hypocotyl response to ACC were obtained: “hypo-insensitivity” lines bore nodule numbers similar to those of WT whereas “hyper-insensitivity” lines, with traits symptomatic of a lack of ethylene response, bore higher nodule numbers (Lohar et al., 2009).

### Detailed Description of the *EIN2* Mutants and Transgenic Plants Altered in Ethylene Perception

*Mtskl* has been characterized in depth and used by many research groups to refine our understanding of the role(s) played by ethylene in nodulation. Typical of hormonal mutants (Karlowski and Hirsch, 2003), *Mtskl* is pleiotropic (Penmetsa et al., 2008; Prayitno, 2010) and the mutation has many effects on the plant (**Table 2**). For example, non-inoculated *Mtskl* exhibit longer primary roots than WT and a delay in lateral root growth in early developmental stage (Prayitno, 2010). Furthermore, *Mtskl* roots are thinner and have longer cortical cells (Prayitno and Mathesius, 2010) and shorter RHs (Oldroyd et al., 2001) than those of non-inoculated WT. *Mtskl* nodulation is root-controlled (Prayitno et al., 2006b) and the mutant roots respond to nitrate although not as much as those of WT (Prayitno, 2010). These two characteristics, root control and nitrate sensitivity, distinguish the hyper-nodulator *Mtskl* from super-nodulators which are affected in autoregulation of nodulation (Novak, 2010). The *Mtskl* mutation also affects nodule positioning because nodules on the mutant are no longer restricted to the cortical zone facing the xylem tissue (Penmetsa and Cook, 1997), where nodules are expected to form since ACO transcripts, indicative of ethylene biosynthesis, are located in phloem-facing cortical tissue (Heidstra et al., 1997). The *Mtskl* mutant is hypersensitive to NFs (Oldroyd et al., 2001) which makes it useful to researchers in the field of transcriptomics as the expression of genes induced by NFs will be up-regulated much more in *skl* than in WT (e.g., Breakspear et al., 2014; Larrainzar et al., 2015). Etiolated seedlings and leaves of *AtEIN2* mutants evolve more ethylene than those of WT (Guzmán and Ecker, 1990) and one would assume that *MtSkl* does the same. Penmetsa et al. (2008) postulated that *skl* is lacking ethylene-mediated ethylene production.

The nodulation phenotype of *Ljenigma-1*, mutated in LjEIN2, differs from that of *Mtskl* (**Table 2**). *Ljenigma-1* shares several traits with *Mtskl*, such as the lack of a typical triple response or a nitrate-sensitive nodulation, although it is more sensitive to nitrate than its WT (Chan et al., 2013). In contrast to *Mtskl*, *Ljenigma-1* displays a low nodule number, independently of the type of micro-symbionts used as inoculant (Chan et al., 2013). Other contrasting traits are the response of *Ljenigma-1* to mycorrhizal fungi and its nodulation phenotype being controlled by both shoot and root (**Table 2**). In seedlings, *Ljenigma-1* roots evolve five times more ethylene than those of WT while the shoots evolve twice as much; this is likely a result of the ethylene-insensitivity of the plants: since the roots do not sense ethylene, they are making more of it (Chan et al., 2013). Furthermore, in contrast to those of *Mtskl* (Penmetsa et al., 2008), roots and shoots in *Ljenigma1* respond differently to ACC treatments. After an 18 h ACC-treatment of non-inoculated plants, *LjETR1* and *LjEIL3* transcript abundance is greatly increased in roots but not in shoots. This suggests that *Ljenigma-1* roots are ethylene-hypersensitive (Chan et al., 2013). The differences between the two mutants do not seem to lie on the type or location of the mutation. Both mutations are recessive and likely affect the C-domain activity of the EIN2 protein, thus altering its function. Thus, the disparities are difficult to reconcile, especially knowing that transgenic roots carrying RNA interference (RNAi) constructs targeting the two *Lotus EIN2* genes exhibit a hyper-nodulation phenotype (**Table 2**; Miyata et al., 2013). The two genes appear to work together as roots having both genes suppressed bear more nodules than either roots carrying an empty vector or roots carrying only one of the suppressed genes. The greatest density of nodules was also found on those roots with reduced expression of both genes (Miyata et al., 2013). Chan et al. (2013) proposed that in *Lotus*, *EIN2a* is responsible for the triple response and the nodulation phenotype, whereas *EIN2b* is involved in nodulation. The *Ljenigma-1* mutant being mutated in the former gene would not display the triple response phenotype and its roots would still be responsive to ethylene via the latter gene (Chan et al., 2013). Thus, the paradox of *Ljenigma-1* likely rests on EIN2 functional redundancy and the different expression patterns exhibited by the two *EIN2* genes.

Studies of transgenic plants have confirmed the results obtained with *Mtskl*, that ethylene inhibits nodulation. Thus, *L. japonicus* plants containing a vector with a mutated gene coding for an ethylene receptor, either *ETR1* of *Arabidopsis* (Lohar et al., 2009) or *ERS1* of *Cucumis melo* (Nukui et al., 2004), display symptoms of ethylene insensitivity (**Table 2**). For example, the ETR1 lines which were ethylene hyper-insensitive display a larger number of infection events, resulting in a larger number of nodules, than those which were hypo-insensitive. Their nodules were smaller and contained higher number of bacteroids per symbiosome than those of WT; furthermore, in these lines a larger number of nodules formed in between xylem poles (Lohar et al., 2009).

### Sites of Action of Ethylene in Nodulation

In this section, I will describe in detail the roles played by ethylene during the different stages of the rhizobial symbiosis (**Figures 1** and **2**), as these were described in section A. Recently, ethylene has been placed at the forefront of the nodulation process by Larrainzar et al. (2015) who demonstrated that upon rhizobial inoculation, two ethylene-regulatory paths are set. The first path occurs fast (1 hai), is transient, independent of NF perception, and positively controlled by ethylene, while the second one occurs later (6 hai), is dependent on NF perception, and is controlled negatively by ethylene (Larrainzar et al., 2015). Whereas the former is likely part of the defense response, the latter corresponds to the activation of the nodulation programs and affects adversely several hormonal signaling pathways leading the authors to propose a master negative regulatory role for ethylene. Upon inoculation, ACS and ACO are up-regulated, and as a result ethylene is produced in the nodulation zone (Larrainzar et al., 2015). These results confirm the work of many who noted the induction of ethylene biosynthesis by NFs (e.g., Miyata et al., 2013; van Zeijl et al., 2015) and an ethylene production increase early in the symbiosis (Ligero et al., 1986; Suganuma et al., 1995; Lopez-Gomez et al., 2012). In fact, as early as 1986, Ligero et al. (1986) proposed that ethylene was likely to control nodule development, maintenance, and senescence as they measured three peaks of ethylene throughout the nodulation process.

#### Host Immunity

As mentioned earlier, the NF signaling cross-talks with the innate immune signaling early in the rhizobial symbiosis (Gourion et al., 2015; Limpens et al., 2015). When rhizobia are attempting to penetrate the plant epidermis, they are subjected to host immune responses, as are pathogens (Zamioudis and Pieterse, 2012; Gourion et al., 2015). The bacteria synthesize several molecules involved in warding off the MTI and ETI defense responses that the plant is putting into place. However, in a nodulating plant, it is not yet entirely understood which bacterial molecule is responsible for which event of the defense response. For example, there appears to be some discrepancy upon which symbiotic genes are expressed in response to the potent plant immune response elicitor flg22. Whereas Lopez-Gomez et al. (2012) did not observe an up-regulation of *NIN*, *NSP1* and *NSP2* upon flg22 perception by *L. japonicus*, Nakagawa et al. (2011) reported that their increased expression was done via the activation of CCaMK (**Figure 1**).

Although ROS production is a well-known defense response, in nodulation of *M. truncatula* it does not appear to be induced by ethylene signaling as abundance of transcripts involved in ROS production was similar in ACC-treated *Mtskl* and WT (Prayitno et al., 2006a). There is, nonetheless, agreement on the enhancement of ethylene production being one of the MTI events (star 1 in **Figure 1**). Earlier studies as well as recent ones have reported a transient evolution of the hormone in the early stages of the symbiosis (e.g., for indeterminate nodules, Ligero et al., 1987; for determinate nodules, Suganuma et al., 1995 and Lopez-Gomez et al., 2012). Furthermore, flg22-treated *Lotus* produces more ethylene, exhibits activated MAP kinases (MAPK) 3 and 6, and displays up-regulated expression of defense-related genes such as the TF *WRK33* (Lopez-Gomez et al., 2012), as flg22-challenged *Arabidopsis* does (Nicaise et al., 2009). If one draws parallels between the two model plants, one could suggest that flg22-treated *Lotus*, as flg22-challenged *Arabidopsis* (Nicaise et al., 2009), produces higher levels of ethylene because FLS2-perceived flg22 induces phosphorylation by MAPK3/6, and therefore activation, of ACS (**Figure 3**). The same MAP kinases phosphorylate EIN3 in *Arabidopsis*, protecting it from degradation and allowing it to bind to ethylene-responsive genes (Yoo et al., 2008). One of these genes is *FLS2* itself as EIN3 binds to the primary ET response element in the *FLS2* promoter region (**Figure 3**; Khatabi and Schäfer, 2012). The parallel between defense responses of legumes and *Arabidopsis* can be extended to include *ein2* mutants. For example, *Atein2* mutants, incapable of sensing ethylene and impaired in MTI, exhibit an enhanced susceptibility to pathogens (Khatabi and Schäfer, 2012). As well, *Mtskl* is more susceptible to both *Rhizoctonia solani* and *Phytophthora medicaginis*, with only 10% of its seedlings surviving infection compared to 80% of the WT seedlings, likely because EIN2 regulates the pathogen progression through a positive feedback amplification of ethylene biosynthesis (Penmetsa et al., 2008). If EIN2 is mutated, then EIN3 is not protected from degradation by EBF1/2 (**Figure 3**) and thus cannot bind to the *FLS2* promoter. As a result, MAP kinases do not phosphorylate ACS and ethylene is not produced, allowing pathogen entry.

Ethylene does not act alone in this response; its action is integrated with the action of SA and JA, hormones known to be involved in plant defense responses. Ethylene, together with JA, can activate ERF1, a TF with an AP2-EREBP domain, responsible for triggering PR expression in *Arabidopsis* (Lorenzo et al., 2003). As well, upon rhizobial inoculation of *L. japonicus*, *LjERF1* expression is up-regulated within 24 hai by both ethylene and JA, triggering the transcription of PR10 (**Figure 1**; Asamizu et al., 2008). *LjERF1* is a positive regulator of nodulation since when it is RNAi-silenced, the plants respond to the rhizobia as if they were pathogens; they increase PR expression which leads to nodulation inhibition. Recently, EIN3/EIL1 has been considered as a key node where the three hormones’ signaling pathways interact. Thus in *Arabidopsis*, ethylene stabilizes EIN3 and EIL1 (Merchante et al., 2013), and JA activates their transcription by promoting the degradation of the JAZ proteins known to repress them (**Figure 3**; Zhu et al., 2011). In *Mtskl*, JA is repressed by bacterial inoculation since the JA receptor JAZ2 and a JA biosynthetic gene are down-regulated (Breakspear et al., 2014). As for SA, EIN3/EIL1 inhibits its synthesis because it binds specifically to the promoter of *SID2* (SA Induction Deficient 2), which encodes a SA biosynthetic enzyme, and prevents the full activation of the defense responses (**Figure 3**; Chen et al., 2009).

#### The Common Signal Transduction Pathway

Ethylene by affecting the CSTP places itself at the heart of nodule development. It modulates calcium spikings (star 2, **Figure 1**) by decreasing their frequency as seen in *Mtskl* which exhibits longer periods between calcium spikes than the WT (Oldroyd et al., 2001). For this specific event, ethylene is thought to work antagonistically with JA inhibiting its action on calcium spiking through EIN2; the rapidity with which JA and ethylene interacts to affect calcium spiking suggests a direct crosstalk (Sun et al., 2006). The behavior of these two hormones is different here in the CSTP to that described earlier in the host immune response where ethylene and JA are working synergistically on EIN3/EIL1 (**Figure 3**). This suggests a very fine tuning (temporal and spatial) of each step of the nodulation process.

In interactions where the epidermis is bypassed by the rhizobia, ethylene appears to be promoting nodulation. In the semi-aquatic tropical legume *Sesbania rostrata* infected by *Azorhizobium caulinodans*, when the roots are flooded, ethylene is required for rhizobial infection (D’Haeze et al., 2003). Rhizobia enter these plants by crack-entry at the base of lateral roots, where the cortical cells are directly exposed to the environment. Once the rhizobia have colonized the exposed fissure, NFs trigger ethylene production and together NFs and hormone induce a programmed cell death allowing the rhizobia to progress inter-cellularly through the cortex. Simultaneously, nodule progenitor cells divide to form a NP toward which the bacterial colony grows (D’Haeze et al., 2003). The ethylene requirement was confirmed by pharmacological treatments, whereby no nodules formed with ethylene inhibitors. In this specific case, and in contrast to nodulation in *L. japonicus*, nodule initiation cannot be uncoupled from rhizobial invasion (D’Haeze et al., 2003). In non-flooded conditions, *Sesbania* develops RHs which are used for bacterial colonization, but in this case ethylene is inhibitory to nodulation (Goormachtig et al., 2004), reinforcing the idea of a specific epidermal control by the hormone. Nodulation in *Ljnena* (**Table 1**) is reminiscent to that seen in plants displaying crack-entry; in this mutant, no ITs form and yet some of the nodules are pink (Groth et al., 2010). Furthermore, nodule infection is promoted in flooded conditions, i.e., when ethylene is produced. As for *Sesbania*, *Ljnena* mutants treated with ethylene inhibitors are not infected (star 3, **Figure 1**). The crack-entry trait of *Ljnena* may be an ancient trait shared by common ancestors of *Lotus* and *Sesbania*, two species which belong to the same legume sub-clade (Groth et al., 2010). Before RH colonization evolved, ethylene may have played a stimulatory role in nodulation. Colonization via the RH would have added a check-point to the invasion process. In fact, ethylene is known to act negatively at the boundary epidermis-cortex in this type of infection (Guinel and LaRue, 1992). The question that posed Guinel and Geil (2002) is still of actuality. Is ethylene synthesized by epidermal cells of a higher plant organ? More specifically, is ACO present in epidermal cells? ACO activity is absent in the epidermal cells of mung bean stems and pea (var. Argenteum) leaves, which has led Osborne (1991) to question the existence of an alternative ethylene pathway in the epidermis of higher plants. Larrainzar et al. (2015) localized ACS transcripts in the epidermis of inoculated and non-inoculated roots; unfortunately, they did not elaborate on the localization of ACO transcripts. Similarly, no mention is made of ethylene biosynthesis genes in the RH “infectome” transcriptomics study of Breakspear et al. (2014). To put at rest this question, it is essential that we ascertain the existence of ACO activity in the legume epidermis.

#### The Epidermal Program

Ethylene negatively influences the epidermal program. Thus, infection events are inhibited by ACC but promoted by AVG in WT (Oldroyd et al., 2001), but they are promoted in ethylene-insensitive plants since these exhibit not only higher infection events but also more ITs than control plants (Penmetsa and Cook, 1997; Nukui et al., 2004; Lohar et al., 2009). ACS expression in RHs displaying aborted ITs suggests that ethylene synthesis may be directly linked to the infection arrest (Larrainzar et al., 2015). However, if epidermal ACO activity is non-existent, then this would mean that ACC itself acts as a signal, in this case inhibitory, which is an interesting concept raised by Van de Poel and Van Der Straeten (2014). Ethylene plays a role as early as 6 hai when RHs are deforming and branching as it regulates the expression of NF-dependent genes involved in actin and tubulin reorganization (star 4, **Figure 1**; Larrainzar et al., 2015). To determine if there is a direct link between ethylene and the cytoskeleton in the process of nodulation, it would be useful to test the ethylene-sensitivity of cytoskeleton-altered mutants, i.e., *Ljnap1, Ljpir1* and *Ljarpc* (**Figure 1**), as these mutants form few ITs which do not enter the cortex. They are able to form nodules but these are empty and without any anatomical structures (Yokota et al., 2009; Hossain et al., 2012). As for *Ljlot1*, it is ethylene-insensitive in terms of nodulation (Ooki et al., 2005) but in contrast to *Mtskl* it forms very few ITs (**Table 1**). It would thus be interesting to cross *Ljlot1* with *Ljein2* mutants to determine whether the two mutations are epistatic.

As mentioned above, ethylene likely controls the IT entry into the cortex (star 5, **Figure 1**; Guinel and Geil, 2002). Ethylene-treated pea roots exhibit more ITs arrested at the interface epidermis-cortex than non-treated roots (Lee and LaRue, 1992a). Conversely, the low nodulator *Psbrz* treated with ethylene antagonists displays ITs breaching into the cortex when non-treated *Psbrz* has most of its ITs arrested in the epidermis (Guinel and LaRue, 1992). Several pea symbiotic mutants have their ITs halted within the epidermal cell base. *Pssym16* (Guinel and Sloetjes, 2000) and *Pssym15* (Jones et al., 2015) are two such mutants; they are also known to accumulate cytokinins (**Table 1**). Interestingly, *Mtcre1* (Gonzalez-Rizzo et al., 2006) and *Ljhit1-1* (Murray et al., 2007), two mutants defective in cytokinin sensing, have a majority of their ITs also blocked in the epidermis. *Mtcre1* (Plet et al., 2011), *Pssym15* (Jones et al., 2015), and *Pssym16* (Guinel and Sloetjes, 2000) are all ethylene-sensitive since they bear more nodules after AVG treatment; however, only *Pssym16* has its nodule number totally restored by AVG. As cytokinin is known to up-regulate ACS5 (**Figure 3**; Hansen et al., 2009), and as *Mtskl* also exhibits a reduced sensitivity to cytokinin (Penmetsa et al., 2008), the two hormones are likely involved in the IT progression across the inner periclinal wall of the infected epidermal cell. In an attempt to distinguish the effects of ethylene from those of cytokinin, Plet et al. (2011) created *skl cre1* mutants; these double mutants exhibited higher nodule number than *Mtcre1* but lower than WT, suggesting that the mutations are epistatic and that the two pathways, i.e., EIN2-dependent ethylene and CRE1-dependent cytokinin, run in parallel. Ethylene perception is likely at the outset of the cytokinin pathway activation because Larrainzar et al. (2015) demonstrated that within 48 hai, NF-dependent, ET-regulated biosynthetic genes of numerous hormones, including cytokinin, are expressed. For cytokinin biosynthesis, ethylene perception is required as transcripts of *MtIPT*, a cytokinin biosynthetic enzyme, are reduced in *Mtskl* (Larrainzar et al., 2015). These results are in agreement with Penmetsa et al. (2008) suggestion that ITs in the epidermis are negatively regulated by cytokinin-induced ethylene perception.

#### The Cortical Program

Because *Mtskl* bear numerous but small nodules, Xiao et al. (2014) proposed that in *M. truncatula* ethylene signaling has a different effect on NP and NM; whereas it would inhibit NP formation, it would strongly promote NM development. However, ethylene would likely not act alone. Thus ethylene is known to control negatively the cortical program by interfering with cytokinin signaling. This is seen with the *L. japonicus* spontaneous nodules which formed in the absence of rhizobia. Treating with AVG either *Ljsnf1* (star 6, **Figure 1**) or *Ljsnf2-2* (star 7, **Figure 1**), a mutant of *CCaMK* and a mutant of *LHK1*, respectively, increases pseudo-nodule number whereas treating the same mutants with ACC decreases that number dramatically (Tirichine et al., 2006b). The response of the ACC-treated mutants inoculated with *M. loti* suggests that their NF-induced ethylene signaling is turned on and that ethylene plays a role in nodule formation downstream of cytokinin perception (Tirichine et al., 2006b). However, this is likely not through MtEFD (**Table 1**), a TF known to target *MtARR4* (Response Regulator) and as such linked to the cytokinin signaling pathway, because (1) its gene expression does not differ between ACC-treated, AVG-treated and WT plants and (2) its transcripts are expressed in inoculated *Mtskl* (Vernié et al., 2008). Plet et al. (2011) suggested that ethylene may restrict cytokinin action to the cortical regions facing xylem poles; by doing so, it would have an indirect effect on positioning NP. NFs have been shown to induce local, MtCRE1-independent, cytokinin accumulation which promotes in a MtCRE1-dependent manner the expression of ACS, suggesting that cytokinin signaling promotes ethylene synthesis (star 7, **Figure 2**) in the cortical cells (van Zeijl et al., 2015). However, because *Mtskl* accumulated more cytokinin than WT, van Zeijl et al. (2015) suggested that a negative feedback loop is at play in the cortex, whereby ethylene would keep in check the cytokinin levels of the cortex to prevent further NP from forming.

Ethylene appears to control the position where nodules initiate, the number of nodule foci which initiate, and the NP growth because in ethylene-insensitive transformants numerous nodules formed in front of the phloem and the number of nodule foci and mature nodules increased (Lohar et al., 2009). Ethylene affects also the position of the nodules along the primary root length as Prayitno and Mathesius (2010), by testing the effect of AVG and silver, an antagonist of ethylene action, on the nodulation zone extent, found that AVG lengthens the zone but silver shortens it in WT, whereas in *Mtskl* AVG has no effect but silver reduces the length of that zone significantly. This effect may be mediated by auxin as ethylene was shown to mediate auxin transport from the shoot to the nodulation zone via EIN2. In *Mtskl*, auxin transport is enhanced resulting in high auxin concentrations in the zone where nodules would initiate, likely promoting NP formation; this would indicate an antagonistic action for the two hormones (Prayitno et al., 2006b).

In the mutant *Ljrel3* (for Reduced Leaflet 3), mutated in an ortholog of *AtAGO7* (ArGOnaute), rhizobial infections are reduced compared to WT and fewer nodules form (Li et al., 2014). *Ljrel3* is likely sensitive to ethylene and may be over-producing it because when *Ljrel3* is treated with AVG or silver, nodulation is restored. Inoculated *Ljrel3* mutants exhibit decreased sensitivity to exogenous auxin, increased sensitivity to auxin transport inhibitors, and increased expression of *ARF3* and *ARF4* (Auxin Response Factor), the gene-products of which are known to be specific targets of REL3 (Li et al., 2014). Additionally, LjREL3 is localized in the root and nodule vascular tissues. As REL3 is a key player in the biogenesis of *TAS3* ta-siRNAs [trans-acting s(hort) i(nterfering) RNAs], Li et al. (2014) proposed that the REL3-derived *TAS3* tasiR-ARF pathway regulates auxin response and transport during nodulation, and that this control is mediated by ethylene. I would go further and propose that via the ta-siRNA pathway, auxin and ethylene are working together in NP positioning. This hypothesis is based on the following: (1) mature ta-siRNAs are mobile molecules involved in developmental patterning and thought to traffic for a short distance from the phloem to target cells where they silence gene expression (Chitwood and Timmermans, 2010), (2) the location of the auxin peak levels and that of ethylene production are both important in determining where nodules are formed, and (3) ethylene could mediate such an action because its low solubility in the aqueous environment of the cell would preclude its action over long distances (Penmetsa et al., 2008).

To complete this section on the ethylene effect on the cortical nodulation program, it is interesting to note that *Mtbit1-2*, an ERN1mutant (**Figure 1**), exhibits atypical root development as its cortical cells are shorter and wider than those of WT, with some cells apparently going through programmed cell death since sporadic intercellular spaces form in its cortex (Middleton et al., 2007), all characteristics of ethylene-hypersensitivity. As such, it may be worthy to characterize this mutant in more depth.

#### Nodule Functioning and Senescence

Ethylene is likely to play a role in bacterial release (star 8, **Figure 2**) as the bacteroid number per symbiosome was increased in ethylene-insensitive transgenic *Lotus* (Lohar et al., 2009). Furthermore the hormone must be important for bacteria elongation since for this event to occur, *MtNAC074* transcripts need to accumulate (star 9, **Figure 2**) and this cannot happen if ethylene biosynthesis and signaling are altered in any way (Karmarkar, 2014). Ethylene appears also essential for bacteroid senescence. For this step to take place, ethylene must activate *MtNAC920* (star 10, **Figure 2**) which is a positive senescence regulator; once the TF is up-regulated, it must bind to MtCP2 for the nodule to senesce (Karmarkar, 2014). It is worth mentioning here that several plant mutants exhibit early senescence (Serova and Tsyganov, 2014); however, to my knowledge, none of these mutants have been linked to ethylene.

Here again, ethylene appears to work in concert with other hormones. Puppo et al. (2005) proposed that ABA and ethylene work together to orchestrate this important step where nutrients from both plant cells and rhizobia are recycled. Whereas ABA would guarantee that the nodule defenses are strong to avoid disease and attack, ethylene would trigger remobilization processes (Puppo et al., 2005). However, in their transcriptomics study on nodule senescence, Van de Velde et al. (2006) did not mention any transcripts related to ABA. In that study, JA, GA, and ethylene are highlighted as playing a major role. The role of ethylene is considered as positive since SAMS, ACO, and several ERF TFs are up-regulated. Nevertheless, SAMS transcripts may also have indicated polyamine biosynthesis, especially since spermidine synthetase induction was noted (Van de Velde et al., 2006).

## Nodulation, Ethylene, and Agriculture

Because of space constraint and despite the importance of this topic, I will not enter into detail here. Today, to alleviate the problem of feeding an ever-growing population, alternatives or supplementation to chemical fertilizers are continuously sought after. Thus, in the literature, one can see reports demonstrating the beneficial effect of adding compounds to the soils [e.g., Fe supplementation to soils to enhance symbiotic nitrogen fixation process (Arora et al., 2010); L-methionine addition to improve crop yield (Aziz et al., 2015)]. I would like to give below three examples illustrating that, from my point of view, our knowledge about ethylene and nodulation is still too limited to propose beneficial applications in agriculture, especially since we do not know the effects ethylene has on other rhizosphere microorganisms.

Because ethylene is a negative regulator of nodulation, scientists have used plant growth-promoting bacteria (PGPBs) possessing the enzyme ACC deaminase (**Figure 3**) as a mean of reducing plant ACC, thus preventing plant ethylene evolution. It is thought that ACC deaminase acts not on the ACC pool present in the plant at the time of inoculation but on any ACC molecules synthesized afterward (Gamalero and Glick, 2015). These two different pools of ACC reflect the different peaks of ethylene observed upon rhizobial inoculation of plants (Ligero et al., 1986). Thus ACC deaminase would decrease the later synthesized-ethylene and not the early transient peak of ethylene seen in the host immune response. This means that ACC deaminase would not interfere with the plant defense responses but may allow more nodules to form. Yet, not everyone agrees with this beneficial role of rhizobial ACC deaminase. For example, Murset et al. (2012) studying the *Bradyrhizobium japonicum*-soybean interaction questioned whether or not the true substrate of ACC deaminase is indeed ACC; in their study the bacterial protein uses ACC as well as D-serine, albeit less efficiently. The authors suggested another role for ACC deaminase as they observed that its gene was strongly up-regulated under micro-oxic conditions; they proposed that the enzyme acts in controlling anoxic metabolism and would be important in nodule functioning, rather than in alleviating plant ethylene levels (Murset et al., 2012).

Salt stress is detrimental to nodulation as it can reduce nitrogenase activity by up to 75% that of control plants. Plants respond to such stress by accumulating polyamines, recognized to stabilize cell structure and membranes, scavenge free radicals, and trigger antioxidant metabolism. Supplementation of soils with SA has been considered to help plants withstand such a stress. Working on *M. sativa*, Palma et al. (2013) demonstrated that SA addition ahead of salt treatment greatly improves plant growth and allows full recovery of nitrogenase activity. Thus, no polyamines accumulated in the nodules and lipid peroxidation enzymes were up-regulated. Furthermore, nodular ACC levels were increased significantly, suggesting that ethylene production was increased. The authors suggested that the SA-treated plants alleviate the salt stress by increasing nodule antioxidant metabolism and rerouting SAM into ethylene biosynthesis rather than polyamine synthesis (Palma et al., 2013). Unfortunately, the authors did not elaborate on how the plant and its nodules dealt with the ethylene thus synthesized.

Finally, as the rhizosphere is a complex environment, supplementing fertilizers with compounds interfering with ethylene evolution may have a positive effect on the rhizobia but a negative effect on other microorganisms. For example, Jones et al. (2015) studying the mutant *Pssym15* demonstrated that mycorrhizal fungi and rhizobia appear to be controlled differently as they enter the root cortex. In that pea mutant, whereas bacterial entry is negatively affected at the epidermal-cortical interface, fungal entry appears to be promoted. As mentioned before, it is thought that ethylene interacts with cytokinin at that interface. So if we were to alter the ethylene levels of the plant in agricultural soils to improve nodulation, we may interfere in a detrimental manner with other microorganisms such as the mycorrhizal fungi. To transfer our knowledge to the field, we must make a shift in our way of thinking, so that we consider the entire ecosystem and not just one organism.

## Conclusion

Here, I have described many studies which demonstrate that ethylene is crucial for the proper development of the rhizobia-legume mutualism. Yet this hormone does not act alone. NFs made by the rhizobia create an upheaval in the root; once they are perceived, ethylene biosynthesis and signaling are induced resulting in the activation of enzymes responsible for the synthesis of many hormones such as auxin (Breakspear et al., 2014), cytokinin, GA, ABA, and strigolactones (Larrainzar et al., 2015). Ethylene has been considered for several decades as a negative regulator for both early and late stages of nodulation. Now we are discovering that it plays positive parts in the symbiosis as it up-regulates transcription at certain steps of the process. Alone or in concert with other hormones, it is involved in host immune responses, nodule organogenesis, nodule positioning, bacterial differentiation, and nodule senescence.

As final remarks, I would like to highlight some of the directions which could be taken to advance this field of study. First and foremost, we must create more ethylene-related legume mutants. To my knowledge, apart from *Mtskl*, there are two legume mutants which overproduce ethylene. Both *Pssym17* and *Psna-1* mutants, displaying few and tiny nodules, produce twice as much ethylene as their WT (Lee and LaRue, 1992b, and Ferguson et al., 2011, respectively). With new genomic tools available, it may be time to look at these two mutants again. Second, it may be worthy to target specific ethylene-related genes, i.e., *ACS5*, *CTR1*, and *EIN3*, in model legumes, so that mutants could be created for in-depth characterization to extend our views beyond EIN2. Third, when phenotyping mutants, we should use a common “template” and measure the same traits so that useful comparisons can be made. Using Xiao et al.’s (2014) fate map as a tool would also be valuable. Fourth, transcriptomic studies should be accompanied whenever possible by proteomics studies as post-transcription controls are abundant. Although not related to ethylene, a good example for the necessity of performing protein tests (proteomics, enzyme activity, etc…) is given by Lang and Long (2015) who mentioned two mutants with white nodules exhibiting leghemoglobin transcript levels similar to those of WT. Finally, as many unsuspected hormonal cross-talks are being uncovered in transcriptomics studies, it is becoming essential that these are complemented by in-depth hormonal studies where the hormones in question, foremost ethylene, are measured. Each experiment should comprise well-designed controls so that it is ensured that the effects reported in the mutants/treated plants are only those directly linked to ethylene.

## Author Contribution

FG is the sole author of this manuscript.

## Conflict of Interest Statement

The author declares that the research was conducted in the absence of any commercial or financial relationships that could be construed as a potential conflict of interest.
